# Supplementary material for: Prevalence of diversified antibiotic resistant bacteria within sanitation related facilities of human populated workplaces in Abbottabad
Source: PLoS One. 2020 Aug 5;15(8):e0233325. doi: 10.1371/journal.pone.0233325 (PMC7406079; doi:10.1371/journal.pone.0233325)

Supplementary Information

Prevalence of Diversified Antibiotic Resistant Bacteria within Sanitation Related Facilities of Human Populated Workplaces in Abbottabad

Jawad Ali[1], Malik Owais Ullah Awan[2], Gulcin Akca[3], Iftikhar Zeb[1], Bilal AZ Amin[2], Rafiq Ahmed[1], Muhammad Maroof Shah[1], Rashid Nazir[2*]

[1]Department of Biotechnology, COMSATS University Islamabad (CUI), Tobe Camp, University Road, postal code 22060, Abbottabad Campus, KPK Pakistan.

[2]Department of Environmental Sciences, COMSATS University Islamabad (CUI), Tobe Camp, University Road, postal code 22060, Abbottabad Campus, KPK Pakistan.

[3]Department of Medical Microbiology, Faculty of Dentistry, Gazi University Ankara, 8.cad.82.sok.No:4 06510 Emek Çankaya Turkey.

[*]Correspondence:

Rashid Nazir, PhD

rashidnazir@cuiatd.edu.pk

Keywords: Hygiene, sanitation, antibiotics, antibiotic resistance, workplaces, antibiotic resistant bacteria

Supplementary table 1: One-way ANOVA for the antibiotics used to evaluate the bacterial susceptibility

ource	DF	SS	MS	F	P
Factor	7	18.755	2.679	12.77	0.000
Error	552	115.843	0.210		
Total	559	134.598			

Supplementary table 2: Antibiotics grouping, based on bacterial resistance, via using Tukey's Method. Means that do not share a letter are significantly different. 4 colors represent 4 different clusters/ groups of antibiotics

	N	Mean	Grouping
Ampiclox	70	0.6714	A
Septran DS	70	0.6286	A B
Amoxicillin	70	0.4429	A B C
Gentamicin	70	0.4143	B C
Clarithro	70	0.4000	B C
Augmentine	70	0.3857	C
Azithromycin	70	0.1429	D
Amikacin	70	0.1286	D

Supplementary table 3: Different bacterial morphotypes and their characteristics, observed in this work.

S. No	Origin	Colony shape	Elevation	Margins
1	TMP	Circular	Convex	Undulate
2	TMP	Irregular	Raised	Undulate
3	TMP	Irregular	Flat	Curled
4	TMP	Circular	Flat	Entire
5	TFP	Irregular	Convex	Curled
6	TFP	Filamentous	Flat	Wavy
7	CMP	Irregular	Flat	Wavy
8	CMP	Filamentous	Raised	Entire
9	CMP	Oval	Flat	Wavy
10	CMP	Irregular	Convex	Wavy
11	CFB	Irregular	Flat	Curled
12	CFP	Irregular	Raised	Curled
13	TFB	Circular	Raised	Wavy
14	HMB	Irregular	Convex	Undulate
15	HMB	Oval	Flat	Curled
16	TFP	Irregular	Raised	Wavy
17	HFP	Filamentous	Flat	Wavy
18	CFP	Irregular	Convex	Undulate
19	HMP	Circular	Raised	Curled
20	HMP	Oval	Flat	Curled
21	HFP	Oval	Convex	Curled
22	HFB	Circular	Raised	Wavy
23	TMB	Oval	Convex	Curled
24	TFB	Irregular	Flat	Wavy

Supplementary table 4: Sanitation related MDR bacterial strains isolated from populated human workplaces and their antibiotic resistance profile for eight different commonly used antibiotics. The column indicates the number of different antibiotics to which the selected bacterial strains showed no compromise for their growth (means resistant). Red means resistance and green mean the susceptibility of bacterial strains for that particular antibiotic.

S. No	Strain code	Origin	Co-amoxyclav	Ampicillin	Amoxicillin	Clarithromycin	Azithromycin	Amikacin	Gentamicin	Co-trimoxazole	MDR to AR
1	HFP7	ATH Female Pot	Green	Red	Red	Red	Green	Green	Green	Red	4
2	HFP11	AMC Male Basin	Green	Red	Red	Red	Red	Green	Green	Red	5
3	TMP16	AMC Male Pot	Green	Red	Red	Green	Green	Green	Green	Red	3
4	HMB17	ATH Male Basin	Red	Red	Red	Red	Red	Red	Red	Red	8
5	TMP19	ATH Female Pot	Green	Red	Red	Red	Green	Green	Green	Red	4
6	CMP21	COMSATS Male Pot	Green	Green	Green	Red	Red	Green	Green	Red	3
7	CFB24	COMSATS Female Basin	Red	Red	Red	Red	Green	Green	Green	Red	5
8	TMB25	ATH Male Basin	Green	Red	Red	Green	Green	Red	Green	Red	4
9	CMP26	COMSATS Male Pot	Green	Red	Red	Green	Green	Red	Red	Red	5
10	CMP28	COMSATS Male Pot	Green	Red	Red	Green	Green	Red	Red	Green	4
11	TFB31	AMC Female Basin	Red	Red	Red	Green	Green	Green	Green	Red	4
12	TMB35	AMC Male Basin	Red	Red	Red	Red	Green	Green	Red	Red	6
13	CMB40	COMSATS Male Basin	Green	Red	Green	Green	Green	Green	Green	Green	1
14	HMP46	ATH Male Pot	Red	Green	Red	Red	Red	Red	Red	Green	6
15	HMP47	ATH Male Pot	Red	Red	Red	Red	Green	Green	Red	Green	5
16	TMB51	AMC Male Basin	Red	Red	Red	Red	Green	Green	Red	Green	5
17	HMB55	ATH Male Basin	Green	Red	Green	Green	Green	Green	Red	Green	2
18	HMP60	ATH Male Pot	Red	Red	Red	Green	Green	Green	Red	Green	4
19	HMB64	ATH Male Basin	Red	Red	Green	Red	Green	Green	Red	Red	5
20	TFP65	AMC Female Pot	Red	Red	Red	Red	Green	Red	Red	Red	7
21	TMB67	AMC Male Basin	Green	Red	Red	Red	Green	Green	Green	Red	4
22	HMB69	ATH Male Basin	Red	Red	Green	Green	Green	Green	Green	Green	2
23	HFP70	ATH Female Pot	Red	Red	Red	Red	Red	Red	Green	Green	6

Supplementary Figure 1 – Number of bacterial morphotypes observed in different sampling points and bacteria selected for the assessment of antibiotic resistance potential against different antibiotics of common therapeutic use.
C, COMSATS University; T, Ayub teaching medical college; H, Ayub teaching hospital; M, male; F, female; B, washbasin; P, sanitary pot.

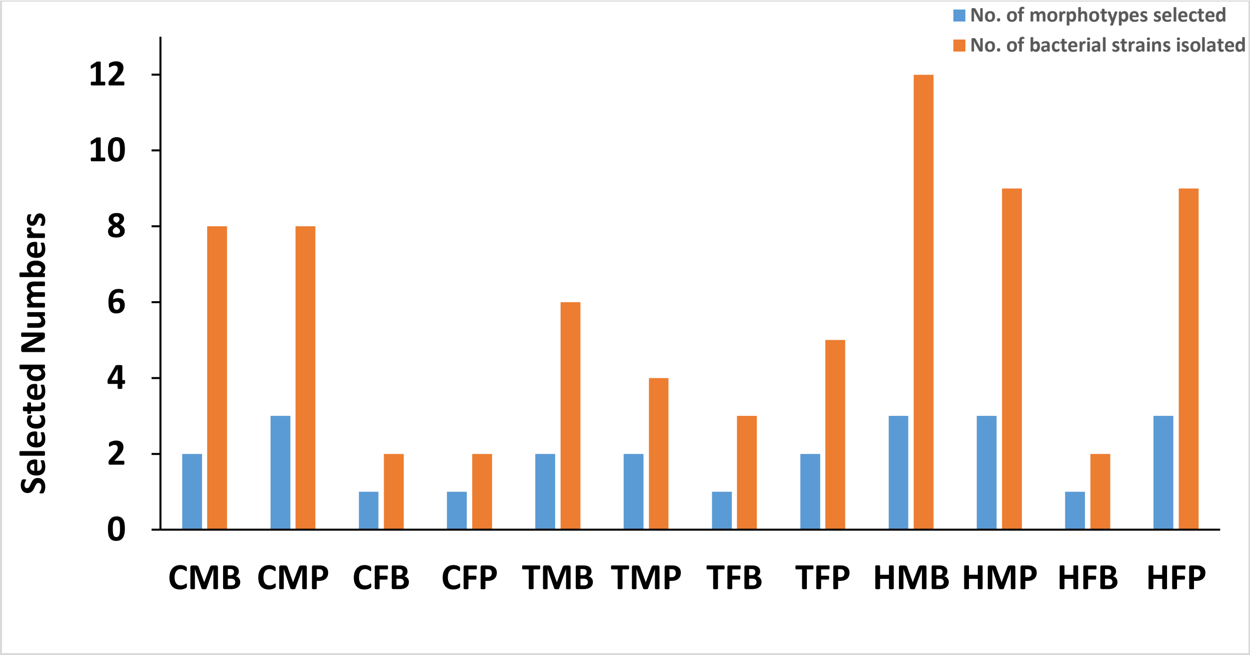

Supplementary Figure 2a – Dendrogram, based on the complete linkage of correlation coefficients' distances, showing the clustering patterns of variables (antibiotics) used in this study

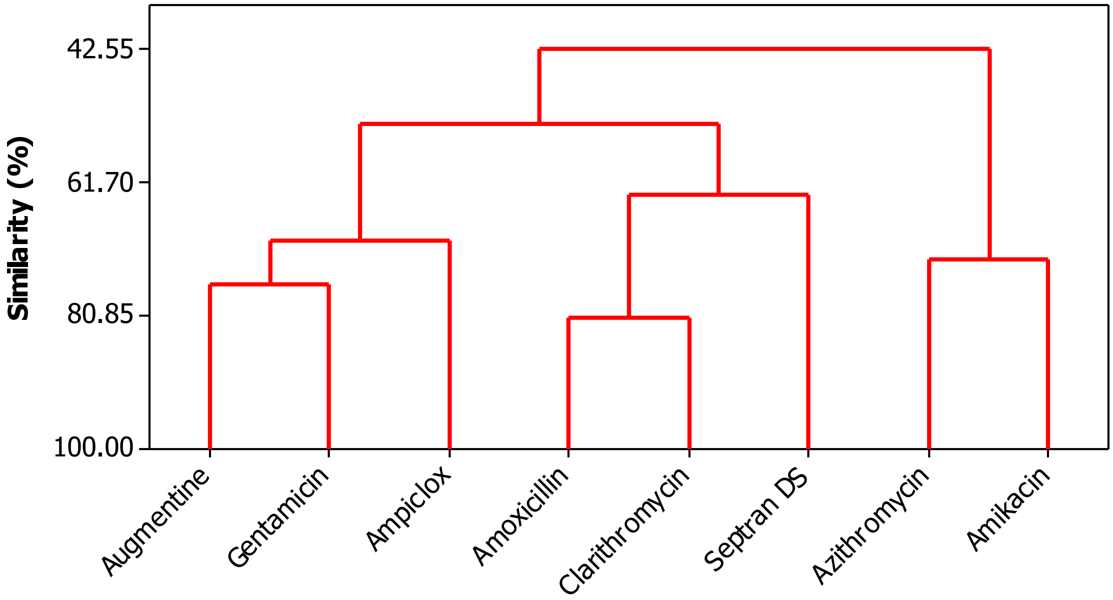

Supplementary Figure 2b – Bi-plot showing the interlinkages between antibiotics used as variables

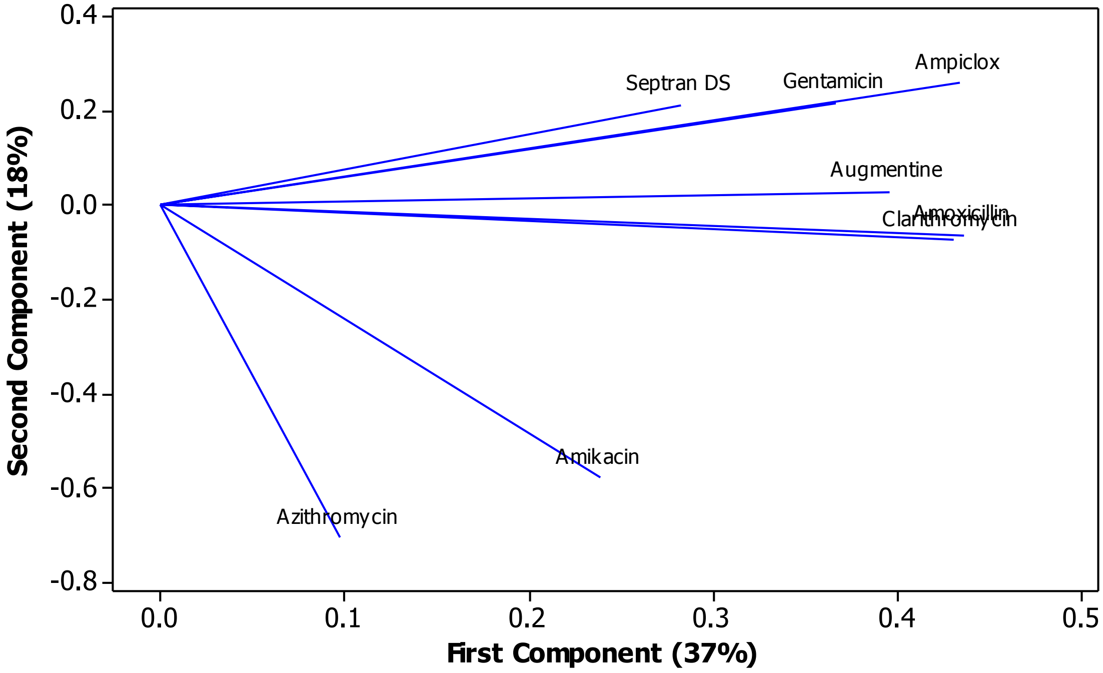

Supplementary Figure 3 – Principle component analysis (PCA), bifurcating the bacterial strains into some loose groups based on their antibiotics' resistance profile.

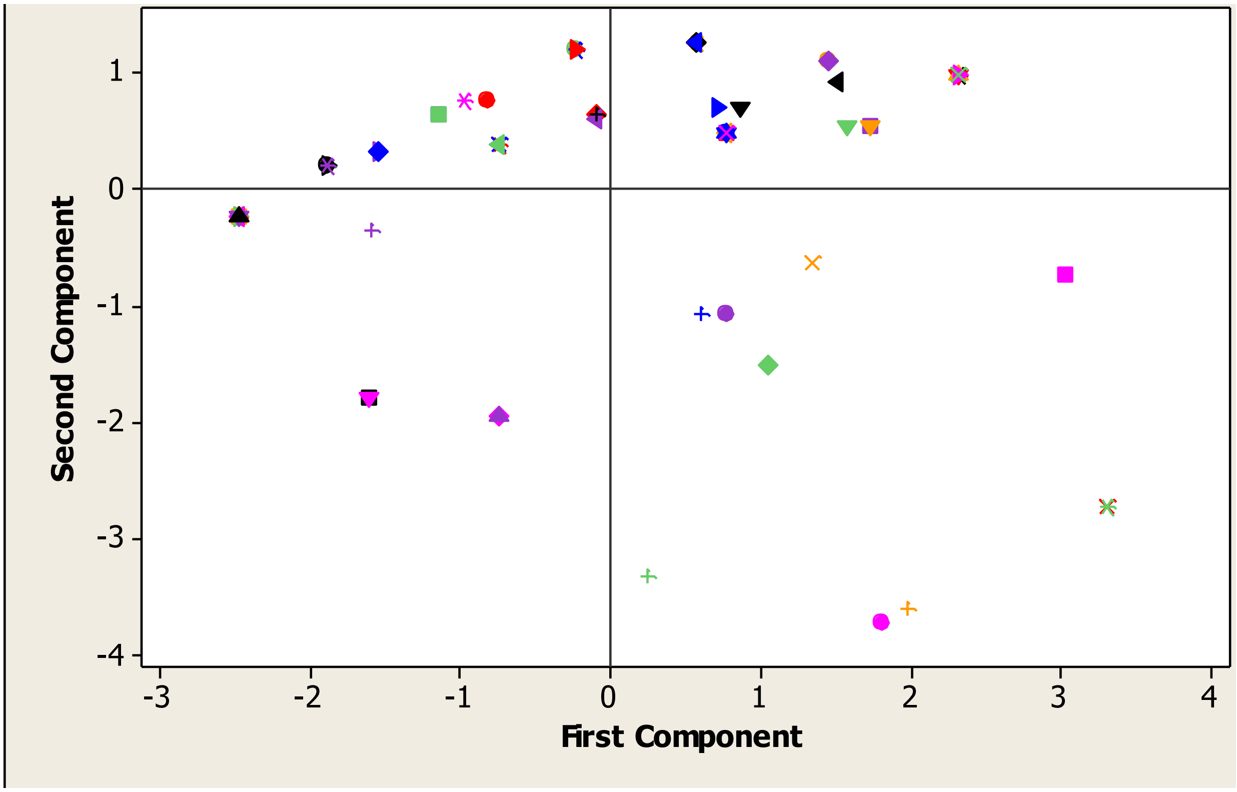

Supplement: S1 File — (PDF) [file pone.0233325.s001.pdf]
